# The association of systemic inflammatory indices with all-cause mortality risks in patients with COPD: A cohort study based on machine learning

**DOI:** 10.1097/MD.0000000000048582

**Published:** 2026-05-01

**Authors:** Lan Cui, Yichao Zhu, Haohao Qian, Hongyang Xu

**Affiliations:** a Department of Critical Care Medicine, The Affiliated Wuxi People’s Hospital of Nanjing Medical University, Wuxi, China.

**Keywords:** chronic obstructive pulmonary disease, machine learning, prognostic model, systemic inflammatory markers

## Abstract

This study aimed to comprehensively assess the prognostic value of routinely obtained blood-based systemic inflammatory indices in predicting all-cause mortality among individuals with chronic obstructive pulmonary disease (COPD). This retrospective cohort study analyzed data from the National Health and Nutrition Examination Survey (NHANES) cycles 2007–2010. A total of 1109 eligible adults with COPD were included, with 333 deaths recorded during the follow-up period. Eleven systemic inflammatory indices were derived from baseline hematological parameters. The associations between these indices and all-cause mortality were initially evaluated using multivariate Cox proportional hazards models. To manage high-dimensional data and identify complex patterns not captured by conventional statistical methods, machine learning (ML) algorithms were applied for feature selection, model development, and performance evaluation. Model discrimination and clinical utility were assessed using the area under the receiver operating characteristic curve (AUC), calibration plots, and decision curve analysis. Among the 1109 participants (mean age 57.9 ± 15.5 years, 52.2% male), non-survivors (n = 333) were significantly older and had a higher baseline burden of comorbidities. After full adjustment for covariates, several inflammatory indices showed statistically significant associations with all-cause mortality. The neutrophil percentage-to-albumin ratio (NPAR) and neutrophil-to-lymphocyte ratio exhibited the strongest associations, with HRs of 2.46 (95% CI: 1.64–3.69) and 2.14 (95% CI: 1.42–3.22), respectively, in the highest quartile (Q4) compared to the lowest (Q1). The neutrophil-to- high-density lipoprotein ratio also demonstrated a significant positive association (HR for Q4 vs Q1: 1.79, 95% CI: 1.18–2.70). In contrast, higher levels of the C-reactive protein-albumin-lymphocyte index index were associated with reduced risk, indicating a protective effect (HR for Q4 vs Q1: 0.49, 95% CI: 0.33–0.72). The ML–derived NPARTEST model, based on the NPAR index, achieved an AUC of 0.828 for predicting all-cause mortality, demonstrating good discriminative performance and clinical utility. Systemic inflammatory indices, particularly the NPAR and neutrophil-to- high-density lipoprotein ratio, are independently associated with all-cause mortality in patients with COPD, often exhibiting nonlinear relationships. The ML–based NPARTEST model demonstrates promising predictive performance. These findings underscore the potential of cost-effective, routinely measured blood-based biomarkers to enhance risk stratification in COPD management. External validation in diverse populations is warranted to confirm the generalizability of these results.

## 1. Introduction

Chronic obstructive pulmonary disease (COPD) is a chronic respiratory condition characterized by persistent airflow limitation, mainly caused by smoking, air pollution, and occupational exposures.^[1,2]^ According to estimates from the World Health Organization, the global COPD patient population reached approximately 328 million in 2020. In 2019, COPD ranked as the third leading cause of death worldwide, accounting for about 3.2 million deaths – trailing only ischemic heart disease and stroke – and contributing to 6% of total global mortality.^[3]^ COPD places a heavy burden on healthcare systems, with frequent acute exacerbations and comorbidities leading to repeated hospitalizations and reduced quality of life.^[4]^ Therefore, identifying a simple, cost-effective biomarker to predict survival and prognosis is crucial for early risk assessment and targeted intervention.

The pathophysiological hallmark of COPD is chronic inflammation of the airways and lung parenchyma, triggered by harmful exposures such as cigarette smoke. This process involves infiltration of neutrophils, macrophages, and CD8^+^ T cells, which release pro-inflammatory mediators like interleukin (IL)-8 and tumor necrosis factor-α.^[5]^ These mediators cause airway mucosal injury, mucus hypersecretion, smooth muscle hyperplasia, and alveolar destruction (emphysema), leading to irreversible airflow limitation, progressive lung function decline, and disease progression.^[6]^ Importantly, this inflammation extends beyond the lungs through circulating cytokines (like tumor necrosis factor-α, IL-6), resulting in systemic low-grade inflammation. This contributes to extrapulmonary comorbidities – such as muscle wasting, cardiovascular disease, and osteoporosis^[7-9]^ – and increases the risk of exacerbations,^[10]^ reduces quality of life,^[11]^ and raises healthcare use and mortality.^[12]^ Traditional inflammatory biomarkers such as C-reactive protein (CRP) and IL-6 have prognostic value in COPD, but their high cost and limited availability limit use in population screening. In contrast, composite hematological indices derived from routine complete blood count tests – such as neutrophil-to-lymphocyte ratio (NLR),^[13]^ neutrophil-percentage-to-albumin ratio (NPAR),^[14]^ systemic inflammation response index (SIRI),^[15]^ systemic immune-inflammation index (SII),^[16]^ lymphocyte-to-monocyte ratio (LMR),^[17]^ and C-reactive protein-albumin-lymphocyte index (CALLY)^[15,18,19]^ – are emerging as cost-effective alternatives. Pathophysiological investigations into COPD have demonstrated multifaceted crosstalk between inflammatory responses and lipid metabolism dysregulation.^[20]^ Accumulating evidence in recent years suggests that aberrant lipoprotein function may contribute significantly to the pathogenesis and progression of COPD.^[21,22]^ Notably, high-density lipoprotein (HDL) levels have been established as one of the critical prognostic indicators for cardiovascular risk stratification in patients with COPD.^[23]^ Furthermore, HDL-associated inflammatory markers – including the platelet-to-HDL ratio,^[24]^ uric acid-to-HDL ratio (UHR),^[25]^ and lymphocyte-to-HDL ratio^[26]^ – are recognized as potential biomarkers for the early diagnosis and therapeutic intervention of COPD. However, the association between the neutrophil-to-HDL ratio (NHR) and COPD remains poorly characterized, warranting further investigation.

Current evidence on COPD inflammatory biomarkers mainly comes from traditional statistical methods (e.g., Cox regression), which may miss complex nonlinear relationships with mortality risk. Comparative studies of multiple biomarkers in large COPD cohorts are also limited, leaving gaps in understanding their combined prognostic value. Machine learning (ML) offers a new perspective: algorithms like RFs and gradient boosting can handle high-dimensional data, detect nonlinear patterns, and improve prediction by integrating multiple variables.^[27]^ Although ML has shown potential in respiratory medicine for predicting disease outcomes, its application to systemic inflammatory biomarkers and COPD mortality remains understudied.^[28-30]^

This cohort study uses ML to examine the association between systemic inflammatory indices and all-cause mortality in COPD patients. By combining advanced analytics with existing epidemiological and pathophysiological knowledge, it aims to generate precise, data-driven evidence for clinical decision-making and to support interdisciplinary advances in chronic disease management.

## 2. Material and methods

### 2.1. Data sources

This study combined cross-sectional analysis with longitudinal mortality follow-up using data from 2 cycles (2007–2010) of the National Health and Nutrition Examination Survey (NHANES), conducted by the US Centers for Disease Control and Prevention (CDC) and the National Center for Health Statistics (NCHS). NHANES employs a complex, multistage probability sampling design to produce nationally representative estimates for the non-institutionalized US population. The survey protocol was approved by the NCHS Ethics Review Board, and all participants provided written informed consent. This analysis followed the Strengthening the Reporting of Observational Studies in Epidemiology (STROBE) guidelines. Data are publicly available at https://wwwn.cdc.gov/nchs/nhanes/default.aspx.

### 2.2. Study population

**Figure 1.**
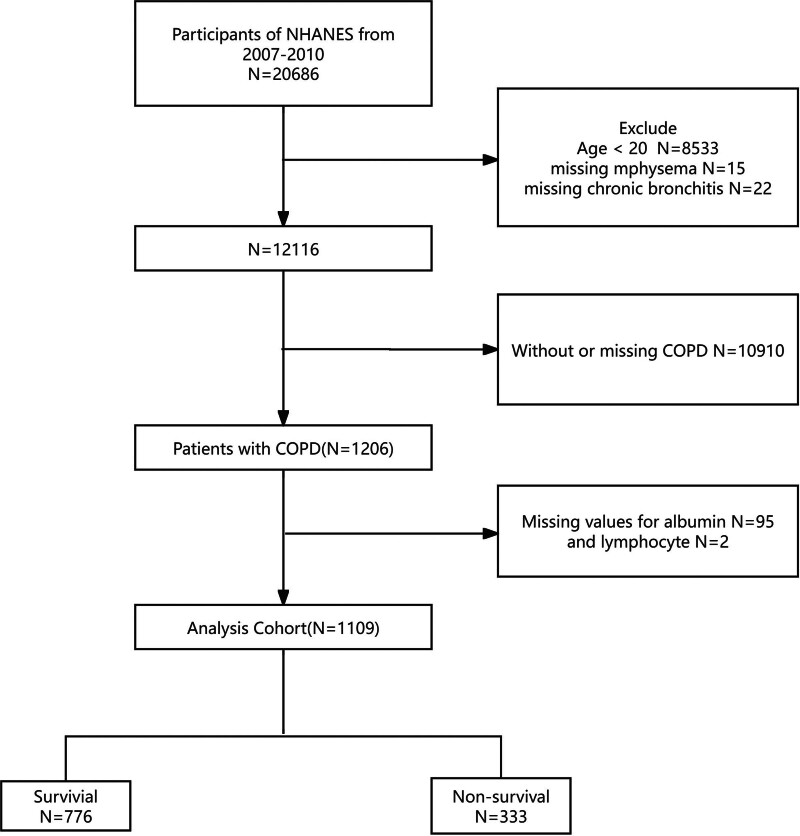
Flowchart of sample selection from NHANES 2007–2010. NHANES = National Health and Nutrition Examination Survey.

The study initially included adult participants aged ≥20 years from the 2007 to 2010 NHANES cycles. COPD was defined based on either: self-reported physician diagnosis of chronic bronchitis or emphysema, or spirometric evidence of airflow limitation after bronchodilator administration (FEV_1_/FVC < 0.7).^[1]^ Participants were excluded if they met any of the following conditions: lacked data on chronic bronchitis or emphysema; had undeterminable COPD status; missing data on key laboratory indicators (serum albumin, lymphocyte count). After applying exclusion criteria, the final analytical cohort comprised 1109 individuals with COPD (Fig. 1).

### 2.3. Variable definitions

#### 2.3.1. Outcome variables

The primary outcome measure was all-cause mortality. Mortality follow-up was ascertained via linkage to the US national death index, where a probabilistic matching algorithm was employed to link NHANES participants to death records. Matching variables included social security number, full name, date of birth, sex, race, and state of residence. Causes of death were categorized using the International Classification of Diseases, 10th Revision (ICD-10) codes, with a primary focus on all-cause mortality (defined as any death recorded with an ICD-10 code). Follow-up duration was calculated from the date of baseline examination to the date of death or December 31, 2019, whichever occurred first.

#### 2.3.2. Systemic inflammation indices

This study assessed eleven systemic inflammation indices derived from routine peripheral blood tests and biochemical markers. Complete blood cell counts were measured using the Beckman Coulter DxH 800 instrument with automated dilution and mixing, and all samples were analyzed at the NHANES mobile examination center. HDL-C was measured via the direct selective clearance method, adhering to NHANES standardized laboratory protocols. During the 2007 to 2010 survey cycles, blood samples were collected, processed, and shipped to the University of Minnesota (Minneapolis) for analysis. All laboratory procedures followed NHANES standardized protocols with strict quality control. Systemic inflammation markers were calculated using untransformed raw data. Each inflammatory index was categorized into quartiles (Q1–Q4) based on prior methods, with Q1 as the reference group. The formulas used to compute these indices are detailed below:

1. NLR: NLR = neutrophil count/lymphocyte count.
2. PLR: PLR = platelet count/lymphocyte count.
3. SII: platelet count × neutrophil count/lymphocyte count.
4. SIRI: SIRI = neutrophil count × monocyte count/lymphocyte count.
5. Aggregate inflammation systemic index (AISI): AISI = neutrophil count × monocyte count × platelet count/lymphocyte count.
6. LMR: LMR = lymphocyte count/monocyte count.
7. CALLY index: CALLY index = C - reactive protein × albumin/lymphocyte count.
8. NHR: NHR = neutrophil count/high-density lipoprotein cholesterol.
9. Monocyte-to-HDL cholesterol ratio (MHR): MHR = monocyte count/high-density lipoprotein cholesterol.
10. NPAR: NPAR = (neutrophil percentage in white blood cell differential count/albumin [g/dL]) × 100.
11. Uric acid-to-HDL ratio (UHR) = serum uric acid (mg/dL)/HDL-C(mg/dL).
*Note: neutrophil percentage refers to the proportion of neutrophils in the white blood cell differential, expressed as a percentage (%).

#### 2.3.3. Covariates

The study included the following covariates: age, sex, race, body mass index, education level, marital status, poverty income ratio (PIR; ≤1.30, 1.30–3.50, >3.50), alcohol consumption, smoking history, hypertension, cardiovascular disease, and cancer. Alcohol consumers were defined as those reporting at least 12 drinks/yr; smokers as individuals with a lifetime consumption of over 100 cigarettes. Hypertension was defined as systolic blood pressure ≥ 140 mm Hg or diastolic blood pressure ≥ 90 mm Hg. Diabetes was identified by self-reported physician diagnosis, HbA1c ≥ 6.5%, fasting plasma glucose ≥ 7.0 mmol/L, or current use of diabetes medication. Cardiovascular disease was defined as a history of congestive heart failure, coronary heart disease, angina, or myocardial infarction as diagnosed by a healthcare professional. Cancer was defined by a positive response to the question: “Have you ever been told by a doctor or health professional that you have cancer or any malignant tumor?”

### 2.4. Statistical analysis

All analyses were conducted by incorporating sample weights to account for the complex sampling design of NHANES, thereby generating nationally representative estimates. All statistical analyses were performed using R software (version 4.5.2) and Python (version 3.9). Statistical significance was defined as a 2-sided *P*-value < .05.

#### 2.4.1. Baseline characteristics and survival analysis

Baseline characteristics of the overall cohort were summarized and stratified by survival status. Group comparisons were conducted using weighted Student’s *t* tests for normally distributed continuous variables, weighted Mann–Whitney *U* tests for skewed continuous variables, and weighted chi-square tests for categorical variables, as appropriate. The association between inflammatory indices – categorized into tertiles – and all-cause mortality was assessed using Kaplan–Meier survival curves and the log-rank test. Multivariable Cox proportional hazards regression models were used to estimate hazard ratios and corresponding 95% confidence intervals. Three models were sequentially adjusted: model 1 was unadjusted; model 2 was adjusted for age, sex, and race; and model 3 was further adjusted for smoking status, body mass index, and comorbid conditions (including hypertension, diabetes, cardiovascular disease, and cancer). To investigate potential nonlinear trends, restricted cubic splines with 3 knots (placed at the 10th, 50th, and 90th percentiles of the distribution) were fitted in Cox models to assess the dose–response relationship between inflammatory indices and respiratory-related mortality.

#### 2.4.2. Machine learning prediction model

The queue was randomly divided into a training set (for model development) and a test set (for performance validation) in a 70% to 30% ratio.

Feature preprocessing and selection: Variables with near-zero variance were removed. Missing values in continuous variables were imputed using multiple imputation, while missing values in categorical variables were imputed using the RF algorithm. Feature selection was conducted using a hybrid strategy: combining the Boruta algorithm (for identifying all potential relevant features) with recursive feature elimination (RFE) to select the optimal feature subset; ultimately, only features identified as important by both methods were retained.

Model construction and hyperparameter optimization: 7 ML models were built and compared in the training set, including Naive Bayes, logistic regression, XG Boos, K-nearest neighbors, support vector machine, RF, and light GBM. The hyperparameters of each model were tuned through 5-fold cross-validation combined with grid search to maximize model performance. Enhanced models were constructed by individually adding relevant inflammatory indices to the baseline model.

Model evaluation: model performance was evaluated on an independent test set. Discrimination was quantified by the area under the receiver operating characteristic curve (AUC); calibration was assessed by calibration curves; clinical utility was evaluated by decision curve analysis for net benefit.

#### 2.4.3. Model interpretability

The shapley additive expanations (SHAP) approach was utilized to interpret the top-performing ML model. Feature importance was ranked based on the mean absolute SHAP values. This ranking provides an overall understanding of the relative contributions of different features to the model’s predictions at a global level.

## 3. Results

### 3.1. Baseline information of the participants with COPD

This study included a total of 1109 patients with COPD, with 776 (70.0%) in the survival group and 333 (30.0%) in the non-survival group. Statistically significant differences were observed in most baseline characteristics between the 2 groups (*P* < .05). In Table 1,compared to the non-survival group, patients in the survival group were significantly younger (53.38 ± 15.15 vs 68.37 ± 10.45 years, *P* < .001), less likely to be male (49.10% vs 59.46%, *P* = .002), and more likely to have attained a college education or higher (44.97% vs 31.83%) and to report high household income (26.68% vs 13.51%; all *P* < .001). With respect to comorbidities, the non-survival group exhibited significantly higher prevalences of diabetes (27.93% vs 14.30%), hypertension (63.66% vs 41.88%), cardiovascular conditions – including heart failure (18.02% vs 5.15%) – and emphysema (47.75% vs 13.27%; all *P* < .001). Laboratory analyses revealed that non-survivors had poorer nutritional status, as indicated by lower serum albumin levels (4.05 ± 0.39 vs 4.20 ± 0.33 g/dL, *P* < .001), elevated inflammatory markers such as C-reactive protein (0.88 ± 1.73 vs 0.46 ± 0.78 mg/dL, *P* < .001), and significantly different levels of multiple systemic inflammatory indices.

**Table 1:** Characteristics of COPD patients stratified by all-cause mortality status.

Variable	Overall	Survivor	Non-survivor	*P*-value
	N = 1109	N = 776	N = 333	
Age (yr)	57.88 (15.51)	53.38 (15.15)	68.37 (10.45)	<.001
Gender (*P*%)				.002
Male	579.00 (52.21%)	381.00 (49.10%)	198.00 (59.46%)	
Female	530.00 (47.79%)	395.00 (50.90%)	135.00 (40.54%)	
BMI, n (*P*%)				.688
Normal (<25)	310.00 (27.95%)	211.00 (27.19%)	99.00 (29.73%)	
Overweight (25–30)	373.00 (33.63%)	264.00 (34.02%)	109.00 (32.73%)	
Obese (≥30)	426.00 (38.41%)	301.00 (38.79%)	125.00 (37.54%)	
Race (*P*%)				.042
Mexican American	80.00 (7.21%)	68.00 (8.76%)	12.00 (3.60%)	
Other Hispanic	84.00 (7.57%)	60.00 (7.73%)	24.00 (7.21%)	
Non-Hispanic White	737.00 (66.46%)	503.00 (64.82%)	234.00 (70.27%)	
Non-Hispanic Black	168.00 (15.15%)	116.00 (14.95%)	52.00 (15.62%)	
Other races	40.00 (3.61%)	29.00 (3.74%)	11.00 (3.30%)	
Educationn (*P*%)				<.001
Less than high school	373.00 (33.63%)	231.00 (29.77%)	142.00 (42.64%)	
High school or equivalent	281.00 (25.34%)	196.00 (25.26%)	85.00 (25.53%)	
College or above	455.00 (41.03%)	349.00 (44.97%)	106.00 (31.83%)	
Marriage (*P*%)				0.047
Yes	580.00 (52.30%)	421.00 (54.25%)	159.00 (47.75%)	
No	529.00 (47.70%)	355.00 (45.75%)	174.00 (52.25%)	
PIR, n (*P*%)				<.001
Low (≤1.3)	379.00 (34.17%)	248.00 (31.96%)	131.00 (39.34%)	
Medium (1.31–3.49)	478.00 (43.10%)	321.00 (41.37%)	157.00 (47.15%)	
High (≥3.5)	252.00 (22.72%)	207.00 (26.68%)	45.00 (13.51%)	
Drinking, n (*P*%)	795.00 (76.30%)	579.00 (78.78%)	216.00 (70.36%)	.005
SMOKE, n (*P*%)	807.00 (72.77%)	525.00 (67.65%)	282.00 (84.68%)	<.001
Diabetes, n (*P*%)	204.00 (18.39%)	111.00 (14.30%)	93.00 (27.93%)	<.001
Hypertension, n (*P*%)	537.00 (48.42%)	325.00 (41.88%)	212.00 (63.66%)	<.001
Cardiovascular diseases				
Congestive heart failure, n (*P*%)	100.00 (9.02%)	40.00 (5.15%)	60.00 (18.02%)	<.001
Coronary heart disease, n (*P*%)	103.00 (9.29%)	46.00 (5.93%)	57.00 (17.12%)	<.001
Angina pectoris, n (*P*%)	76.00 (6.85%)	36.00 (4.64%)	40.00 (12.01%)	<.001
Heart attack n (*P*%)	133.00 (11.99%)	54.00 (6.96%)	79.00 (23.72%)	<.001
Emphysema, n (*P*%)	262.00 (23.62%)	103.00 (13.27%)	159.00 (47.75%)	<.001
Chronic bronchitis, n (*P*%)	617.00 (55.64%)	450.00 (57.99%)	167.00 (50.15%)	.016
Cancer, n (*P*%)	175.00 (15.78%)	101.00 (13.02%)	74.00 (22.22%)	<.001
Albumin (g/dL)	4.16 (0.36)	4.20 (0.33)	4.05 (0.39)	<.001
WBC (×10^9^/L)	7.61 (3.16)	7.56 (3.45)	7.75 (2.31)	.040
Hb (g/dL)	14.21 (1.55)	14.31 (1.44)	13.97 (1.76)	.002
PLT (×10^9^/L)	256.96 (74.00)	263.28 (72.00)	242.22 (76.57)	<.001
HCT (%)	41.45 (4.29)	41.69 (3.99)	40.88 (4.88)	.007
RDW (%)	13.20 (1.57)	12.97 (1.28)	13.73 (2.00)	<.001
NEPCT (%)	60.13 (10.22)	59.03 (9.84)	62.70 (10.62)	<.001
LYM (×10^9^/L)	2.14 (2.23)	2.24 (2.59)	1.93 (0.90)	<.001
MONO (×10^9^/L)	0.57 (0.25)	0.56 (0.25)	0.60 (0.24)	.004
NEMO (×10^9^/L)	4.62 (1.78)	4.49 (1.72)	4.93 (1.89)	<.001
CRP (mg/dL)	0.59 (1.17)	0.46 (0.78)	0.88 (1.73)	<.001
HDL (mmol/L)	1.35 (0.45)	1.36 (0.44)	1.32 (0.47)	.029
TC (mg/dL)	195.09 (42.81)	198.44 (41.39)	187.28 (45.05)	<.001
TR (mg/dL)	142.84 (115.52)	142.31 (126.18)	144.06 (85.97)	.360
NPAR, mean (SD)	1460.86 (318.69)	1413.03 (262.56)	1572.33 (400.27)	<.001
UHR, mean (SD)	4.74 (2.25)	4.55 (2.05)	5.20 (2.60)	<.001
LMR, mean (SD)	4.01 (1.92)	4.22 (1.90)	3.51 (1.86)	<.001
SII, mean (SD)	644.76 (433.20)	609.96 (396.39)	725.87 (500.07)	<.001
CALLY, mean (SD)	8.76 (52.59)	10.26 (62.37)	5.26 (11.49)	<.001
MHR, mean (SD)	0.48 (0.32)	0.46 (0.32)	0.52 (0.30)	<.001
AISI, mean (SD)	369.97 (299.30)	340.87 (271.79)	437.79 (346.32)	<.001
NHR, mean (SD)	3.87 (2.12)	3.69 (1.98)	4.29 (2.37)	<.001
NLR, mean (SD)	2.52 (1.50)	2.30 (1.22)	3.04 (1.91)	<.001
SIRI, mean (SD)	1.44 (1.05)	1.28 (0.85)	1.81 (1.34)	<.001
PLR, mean (SD)	137.97 (60.80)	135.13 (57.08)	144.60 (68.35)	.069

AISI = aggregate inflammation systemic index, BMI = body mass index, CALLY index = C-reactive protein-albumin-lymphocyte index, Hb = hemoglobin, HCT = hematocrit, HDL-C = high-density lipoprotein cholesterol, LMR = lymphocyte-to-monocyte ratio, LYM = lymphocyte, MHR = monocyte-to-high-density lipoprotein cholesterol ratio, MONO = monocyte, NENO = neutrophil, NEPCT = percentage of neutrophils, NHR = neutrophil-to-high-density lipoprotein cholesterol ratio, NLR = neutrophil-to-lymphocyte ratio, NPAR = neutrophil percentage-to-albumin ratio, PLR = platelet-to-lymphocyte ratio, PLT = platelet, RBC = red blood cell, RDW = red cell distribution width, SII = systemic immune-inflammation index, SIRI = systemic inflammation response index, TC = total cholesterol, TG = triglyceride, UHR = urea to high-density lipoprotein cholesterol ratio, WBC = white blood cell.

### 3.2. Kaplan–Meier (KM) survival curve

**Figure 2.**
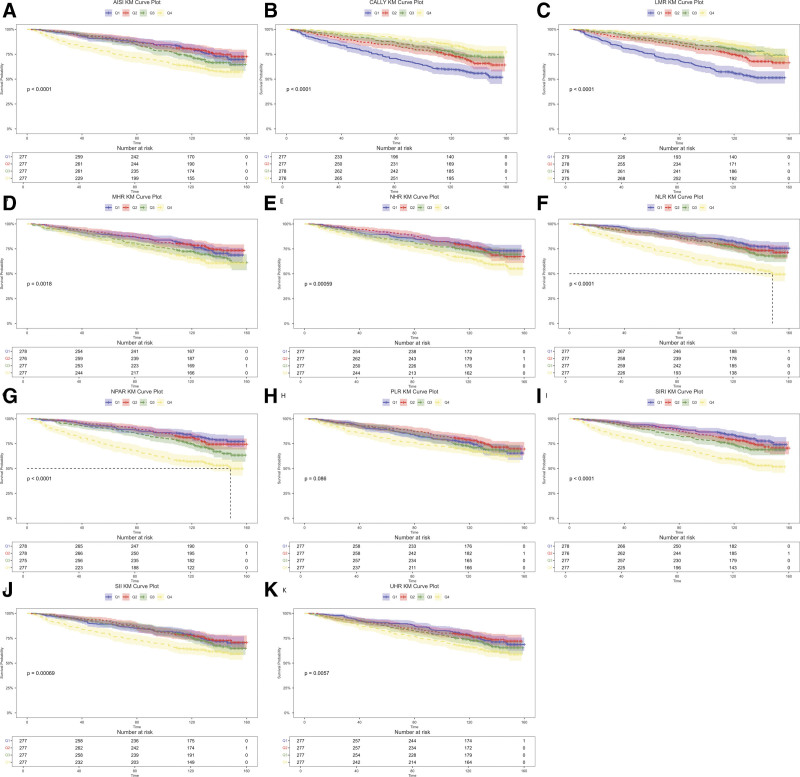
K–M Survival curves for all-cause mortality among systemic inflammatory indices. (A) AISI; (B) CALLY; (C) LMR; (D) MHR; (E) NHR; (F) NLR; (G) NPAR; (H) PLR; (I) SIRI; (J) SII; and (K) UHR. AISI = aggregate inflammation systemic index, CALLY = C-reactive protein-albumin-lymphocyte, LMR = lymphocyte-to-monocyte ratio, MHR = monocyte-to-high-density lipoprotein cholesterol ratio, NHR = neutrophil-to-high-density lipoprotein cholesterol ratio, NLR = neutrophil-to-lymphocyte ratio, NPAR = neutrophil percentage-to-albumin ratio, PLR = platelet-to-lymphocyte ratio, SII = systemic immune-inflammation index, SIRI = systemic inflammation response index, UHR = uric acid-to-high-density lipoprotein ratio.

To evaluate the associations between various inflammatory markers (AISI, CALLY, LMR, MHR, NHR, NLR, NPAR, PLR, SII, and SIRI) and overall mortality, Kaplan–Meier survival analysis (Fig. 2) was utilized to analyze the corresponding survival curves. The results indicated that for AISI, MHR, NHR, NLR, NPAR, SII, and SIRI, all *P*-values were below.001, showing strong statistical significance. The survival curves showed a marked decline in survival probability as the levels of these markers increased. Additionally, both CALLY and LMR were found to be significantly linked to all-cause mortality in individuals with COPD. Notably, lower levels of these 2 markers were associated with higher mortality risk. In contrast, the PLR marker yielded a *P*-value of .096, indicating no statistically significant difference across groups. These findings suggest that, with the exception of PLR, elevated levels of the examined inflammatory markers are independently associated with increased risk of all-cause death.

### 3.3. Cox regression analysis for systemic inflammatory indices and mortality

Multivariate Cox regression (model 3) showed that, compared to the lowest quartile (Q1), the highest quartile (Q4) of several inflammatory markers was significantly associated with all-cause mortality. NPAR and NLR had the strongest associations, with death risk increased by 146.1% (HR: 2.461, 95% CI: 1.643–3.686) and 113.9% (HR: 2.139, 95% CI: 1.421–3.219), respectively. NHR, SIRI, and AISI also showed significant positive associations, increasing risk by 78.6% (HR: 1.786), 61.8% (HR: 1.618), and 52.3% (HR: 1.523), respectively. In contrast, CALLY and LMR were significantly protective: higher levels were linked to 51.4% (HR: 0.486) and 37.4% (HR: 0.626) lower mortality risk, indicating that lower values confer higher risk. After adjusting for covariates, PLR, UHR, and MHR showed no significant independent association with all-cause mortality (all *P* > .05; Table 2).

**Table 2:** Cox regression models for the associations between inflammatory indices and mortality in patients with COPD.

	Model 1^*^		Model 2^*^		Model 3^*^	
Characteristics	Hazard ratio (95% CI)	*P* value	Hazard ratio (95% CI)	*P* value	Hazard ratio (95% CI)	*P* value
All-cause mortality						
NPAR						
Q1						
Q2	1.181 (0.826, 1.689)	.363	1.138 (0.774, 1.675)	.511	1.276 (0.835, 1.951)	.260
Q3	1.624 (1.158, 2.278)	.005	1.318 (0.916, 1.897)	.137	1.513 (0.999, 2.292)	.051
Q4	2.947 (2.146, 4.046)	<.001	2.061 (1.449, 2.933)	<.001	2.461 (1.643, 3.686)	<.001
LMR						
Q1						
Q2	0.541 (0.411, 0.713)	<.001	0.827 (0.605, 1.131)	.235	0.772 (0.552, 1.081)	.132
Q3	0.393 (0.290, 0.532)	<.001	0.692 (0.490, 0.978)	.037	0.679 (0.465, 0.992)	.045
Q4	0.348 (0.255, 0.475)	<.001	0.678 (0.473, 0.973)	.035	0.626 (0.418, 0.939)	.023
SII						
Q1						
Q2	0.969 (0.698, 1.346)	.853	0.816 (0.571, 1.167)	.266	0.941 (0.631, 1.403)	.765
Q3	1.144 (0.834, 1.568)	.404	0.919 (0.654, 1.291)	.627	1.007 (0.685, 1.481)	.973
Q4	1.656 (1.227, 2.235)	.001	1.264 (0.902, 1.772)	.174	1.39 (0.943, 2.049)	.096
CALLY						
Q1						
Q2	0.622 (0.472, 0.821)	.001	0.584 (0.431, 0.790)	<.001	0.559 (0.402, 0.776)	.001
Q3	0.501 (0.374, 0.671)	<.001	0.559 (0.405, 0.771)	<.001	0.514 (0.358, 0.738)	<.001
Q4	0.362 (0.262, 0.499)	<.001	0.478 (0.339, 0.674)	<.001	0.486 (0.331, 0.715)	<.001
AISI						
Q1						
Q2	0.938 (0.667, 1.321)	.715	0.940 (0.647, 1.365)	.744	0.951 (0.626, 1.443)	.812
Q3	1.340 (0.975, 1.843)	.071	1.029 (0.717, 1.477)	.875	1.056 (0.707, 1.578)	.79
Q4	1.915 (1.415, 2.592)	<.001	1.491 (1.046, 2.124)	.027	1.523 (1.018, 2.279)	.041
NHR						
Q1						
Q2	1.106 (0.797, 1.535)	.546	1.076 (0.750, 1.543)	.691	1.231 (0.827, 1.833)	.306
Q3	1.189 (0.860, 1.646)	.295	1.255 (0.866, 1.817)	.23	1.178 (0.78, 1.781)	.436
Q4	1.754 (1.296, 2.374)	<.001	1.679 (1.167, 2.415)	.005	1.786 (1.184, 2.696)	.006
SIRI						
Q1						
Q2	1.249 (0.880, 1.773)	.213	1.090 (0.744, 1.595)	.659	1.07 (0.696, 1.644)	.759
Q3	1.522 (1.084, 2.137)	.015	1.251 (0.860, 1.822)	.242	1.323 (0.877, 1.996)	.183
Q4	2.701 (1.973, 3.696)	<.001	1.613 (1.115, 2.332)	.011	1.618 (1.068, 2.451)	.023
NLR						
Q1						
Q2	1.217 (0.860, 1.722)	.267	1.058 (0.726, 1.543)	.769	1.329 (0.871, 2.03)	.187
Q3	1.373 (0.980, 1.925)	.066	1.157 (0.804, 1.664)	.433	1.4 (0.926, 2.117)	.111
Q4	2.645 (1.939, 3.607)	<.001	1.748 (1.224, 2.495)	.002	2.139 (1.421, 3.219)	<.001
PLR						
Q1						
Q2	0.851 (0.619, 1.170)	.320	0.935 (0.667, 1.311)	.697	1.051 (0.722, 1.53)	.794
Q3	1.032 (0.760, 1.401)	.839	0.915 (0.649, 1.289)	.61	1.019 (0.69, 1.498)	.925
Q4	1.260 (0.937, 1.694)	.127	1.114 (0.804, 1.543)	.518	1.198 (0.831, 1.726)	.334
UHR						
Q1						
Q2	0.956 (0.688, 1.327)	.786	1.197 (0.833, 1.721)	.332	1.01 (0.676, 1.510)	.960
Q3	1.208 (0.885, 1.648)	.234	1.141 (0.801, 1.624)	.465	0.964 (0.649, 1.431)	.854
Q4	1.538 (1.141, 2.072)	.005	1.482 (1.036, 2.120)	.031	1.332 (0.898, 1.977)	.155
MHR						
Q1						
Q2	0.905 (0.648, 1.262)	.556	0.820 (0.567, 1.186)	.292	0.764 (0.512, 1.141)	.189
Q3	1.288 (0.944, 1.758)	.111	1.141 (0.805, 1.618)	.459	1.069 (0.725, 1.576)	.737
Q4	1.543 (1.142, 2.084)	.005	1.242 (0.869, 1.776)	.234	1.110 (0.745, 1.655)	.608

AISI = aggregate inflammation systemic index, CALLY index = C-reactive protein-albumin-lymphocyte index, LMR = lymphocyte-to-monocyte ratio, MHR = monocyte-to-high-density lipoprotein cholesterol ratio, NHR = neutrophil-to-high-density lipoprotein cholesterol ratio, NLR = neutrophil-to-lymphocyte ratio, NPAR = neutrophil percentage-to-albumin ratio, PLR = platelet-to-lymphocyte ratio, SII = systemic immune-inflammation index, SIRI = systemic inflammation response index, UHR = urea to high-density lipoprotein cholesterol ratio.

* Model 1: No covariates were adjusted for.

* Model 2: Adjusted for age, gender, race, marital status, educational level, PIR, smoking, and drinking.

* Model 3: Adjusted for age, gender, race, BMI, educational level, marital status, PIR, smoking, drinking, hypertension, cardiovascular diseases, and diabetes and cancer.

### 3.4. Nonlinear relationships between systemic inflammatory indices and mortality

**Figure 3.**
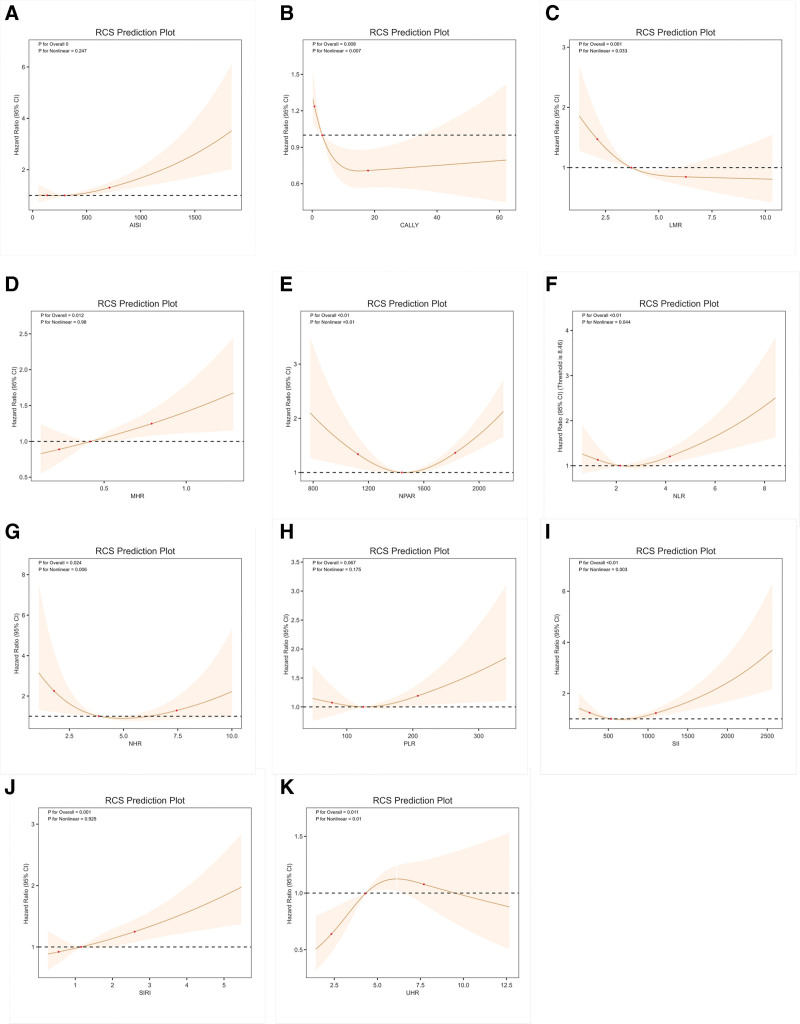
Associations between systemic inflammatory indices and mortality in patients with COPD. (A) AISI; (B) CALLY; (C) LMR; (D) MHR; (E) NPAR; (F) NLR; (G) NHR; (H) PLR; (I) SII; (J) SIRI; and (K) UHR. AISI = aggregate inflammation systemic index, CALLY = C-reactive protein-albumin-lymphocyte, LMR = lymphocyte-to-monocyte ratio, MHR = monocyte-to-high-density lipoprotein cholesterol ratio, NHR = neutrophil-to-high-density lipoprotein cholesterol ratio, NLR = neutrophil-to-lymphocyte ratio, NPAR = neutrophil percentage-to-albumin ratio, PLR = platelet-to-lymphocyte ratio, SII = systemic immune-inflammation index, SIRI = systemic inflammation response index, UHR = uric acid-to-high-density lipoprotein ratio.

The associations between various inflammatory indices and mortality were evaluated using RCS. Several indices demonstrated significant nonlinear relationships: CALLY, LMR, NLR, SII, NPAR, and NHR all showed significant overall associations (*P* for overall < .01) with evidence of nonlinearity (*P* for nonlinear < .05). Among these, NLR, SII, NPAR, and NHR exhibited U-shaped associations, indicating increased risk at both low and high levels. In contrast, AISI, MHR, and SIRI showed significant linear positive associations with mortality (*P* for overall < .01; *P* for nonlinear > .05). PLR was not significantly associated with the outcome (*P* for overall = .067; *P* for nonlinear = 0.175). In summary, most inflammatory indices – except PLR – were significantly associated with respiratory-related mortality, with varying dose–response shapes (Fig. 3).

### 3.5. Feature selection in machine learning

**Figure 4.**
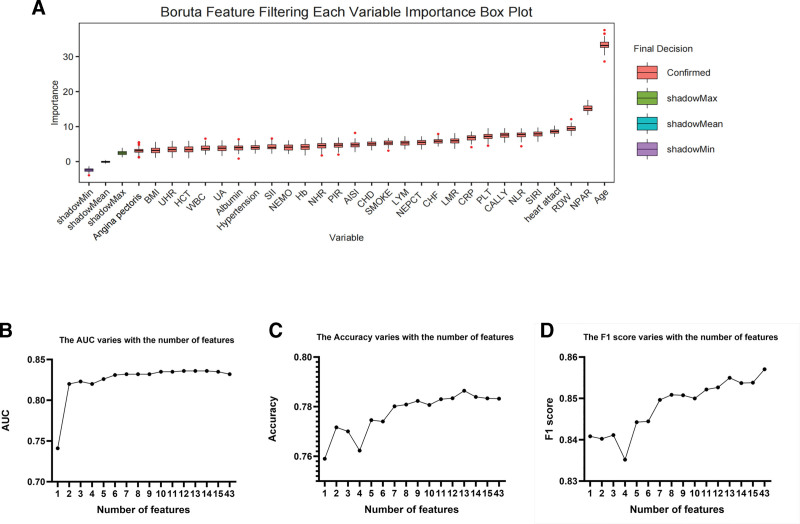
Feature selection for all-cause mortality using the Boruta algorithm and RFE analysis. (A) Boruta feature selection; (B) RFE AUC; (C) RFE accuracy; and (D) RFE F1 score. AUC = operating characteristic curve, RFE = recursive feature.

RFE evaluated the impact of the number of features (ranging from 1 to 43) on model performance by gradually eliminating the least important features. Peak AUC (0.836) occurred at 12 features (accuracy: 0.783; F1: 0.853). Accuracy peaked at 13 features (0.786), with AUC unchanged. F1 peaked at 43 features (0.857), but AUC and accuracy declined slightly (Fig. 4B–D). After balancing performance, RFE selected 13 key variables: SMOKE, SIRI, platelet count, PIR, NPAR, NLR, NHR, heart attack, congestive heart failure, red cell distribution width (RDW), CRP, CALLY, and age. BORUTA, based on the importance assessment of RFs, identified 26 important features (Fig. 4A). The 2 methods jointly selected 12 variables, and the final model included: SMOKE, SIRI, Platelet Count, PIR, NPAR, NLR, NHR, heart attack, coronary heart disease, RDW, CALLY, and Age.

**Figure 5.**
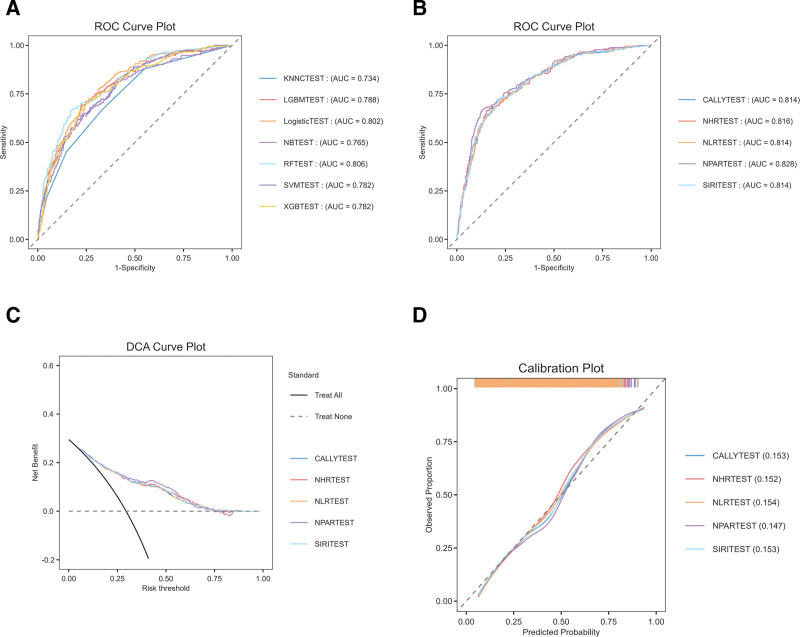
Predictive performance of the original and 5 modified models. (A) baseline model’s training; (B) the ROC of modified models; (C) DCA Curve Plot of modified models; (D) calibration plot of modified models. CALLYTEST: baseline model + CALLY; NHRTEST: baseline model + NHR; NLRTEST: baseline model + NLR; NPARTEST: baseline model + NPAR; SIRITEST: baseline model + SIRI. CALLY = C-reactive protein-albumin-lymphocyte, DCA = decision curve analysis, NHR = neutrophil-to-high-density lipoprotein cholesterol ratio, NLR = neutrophil-to-lymphocyte ratio, NPAR = neutrophil percentage-to-albumin ratio, SIRI = systemic inflammation response index.

SIRI, NHR, NLR, CALLY, and NPAR were confirmed as significant predictors of all-cause mortality in COPD patients. To quantify their relative importance and individual risk contributions, we compared Boruta and RF selection, identifying 15 key variables: age, gender, SMOKE, diabetes, congestive heart failure, coronary heart disease, heart attack, RDW, CRP, lymphocyte, percentage of neutrophils, Albumin, hematocrit, and total cholesterol. These were used to train a baseline model. Seven algorithms were evaluated: Naive Bayes, logistic regression, XGBoost, KNN, SVM, random forest (RF), and LightGBM. The RFTEST model achieved the best performance (accuracy: 0.777; AUC: 0.806) and was selected for developing the improved model (Fig. 5A).

### 3.6. Model development and validation

Separate feature sets were developed for each inflammatory indicator. The diagram demonstrates the enhanced predictive capability of SIRI, NHR, NLR, CALLY, and NPAR in forecasting both all-cause mortality. (Fig. 5B) Adding them individually to the baseline model yielded modest C-statistic gains. Built on the NPAR inflammatory index, the NPARTEST model exceeded the other models based on CALLY, NHR, SIRI, and NLR in discrimination (AUC 0.828), calibration (MCC 0.502), and overall effectiveness. To evaluate clinical utility, decision curve analysis was performed (Fig. 5C–D). For all-cause mortality, integrating systemic inflammatory markers into the baseline model increased net benefit across a wide range of clinically relevant threshold probabilities.

**Figure 6.**
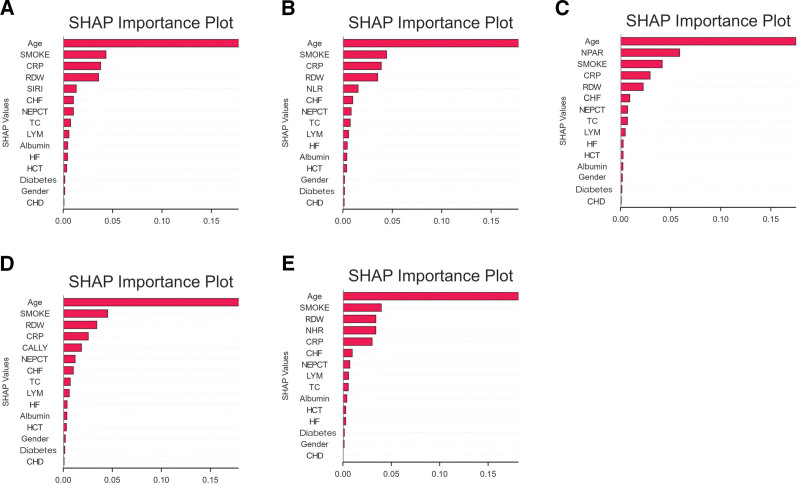
Feature importance of 5 models. (A) SIRI; (B) NLR; (C) NPAR; (D) CALLY; (E) NHR. CALLY = C-reactive protein-albumin-lymphocyte, CHD = coronary heart disease, CHF = congestive heart failure, CRP = C-reactive protein, HCT = hematocrit, HF = heart attack, LYM = lymphocyte, NEPCT = percentage of neutrophils, NHR = neutrophil-to-high-density lipoprotein cholesterol ratio, NLR = neutrophil-to-lymphocyte ratio, NPAR = neutrophil percentage-to-albumin ratio, SIRI = systemic inflammation response index, TC = total cholesterol.

Subsequently, the SHAP model was utilized to interpret and visualize feature importance. In the prediction of all-cause mortality, SIRI, NLR, NHR, CALLY, and NPAR emerged as significant predictors, with NPAR being the key predictor second only to age (Fig. 6).

## 4. Discussion

By integrating ML with blood-based biomarkers, this study delineated independent nonlinear associations between systemic inflammatory markers and all-cause mortality in patients with COPD, thereby offering a novel perspective on prognostic assessment for COPD. The novelty of this study is threefold: methodological breakthrough: For the first time, ML was applied to nonlinear risk modeling of inflammatory markers in COPD, overcoming the constraints of traditional statistical approaches. Biomarker integration: we demonstrated that the neutrophil-platelet-albumin ratio (NPAR) – a composite index capturing the dynamic balance among platelets, neutrophils, and albumin – exhibits superior prognostic value compared to single inflammatory parameters. Additionally, we identified the neutrophil-to-HDL cholesterol ratio (NHR) as a significant predictor of mortality risk in COPD.3) Clinical translatability: The developed NPARTEST model, leveraging routine complete blood count data, offers advantages of low cost and operational simplicity, making it particularly well-suited for COPD management in low- and middle-income countries.

Chronic inflammation plays a pivotal role in the pathogenesis of COPD. While conventional inflammatory markers such as C-reactive protein (CRP) and IL-6 have been linked to elevated mortality in COPD cohorts, the present study underscores the utility of systemic inflammatory indices that capture both inflammatory activity and immune dysregulation.^[31,32]^ Neutrophils, a critical component of the innate immune response, exert key functions in COPD-related inflammatory processes, including degranulation, phagocytosis of viruses or antigens, and release of neutrophil extracellular traps.^[5,33]^ Our findings reveal a nonlinear association between the neutrophil-to-HDL ratio (NHR) and all-cause mortality in COPD patients, with both suboptimal and excessive NHR levels correlating with increased mortality risk.

Prior research has established that HDL serves roles in COPD extending beyond lipid transport. Positioned at the interface of metabolism and innate immunity, HDL profoundly modulates pulmonary inflammation, systemic manifestations, clinical heterogeneity, and comorbidities such as cardiovascular disease in COPD via multiple mechanisms – including regulation of macrophage inflammatory activity, endotoxin neutralization, and transport of bioactive molecules – exhibiting dual anti-inflammatory and pro-inflammatory properties.^[34,35]^ A study by Wen et al demonstrated a nonlinear relationship between HDL levels and COPD: when HDL concentrations fell below 66 mg/dL, elevated HDL was significantly associated with impaired lung function, exacerbated gas trapping, and increased emphysema severity.^[36]^

The dose–response patterns observed among systemic inflammatory indices may also be attributed to their underlying immunopathophysiological mechanisms. In the current study, RCS analysis revealed significant nonlinear associations between the NLR, SIRI, and NPAR with all-cause mortality, whereas CALLY exhibited a monotonic linear relationship. These discrepancies likely reflect the divergent immune processes captured by each index. Neutrophil-based indices (NLR, SIRI, NPAR) integrate markers of innate immune activation with indicators of adaptive immune suppression. For example, NLR combines neutrophil-driven innate immunity with lymphopenia (associated with impaired immune surveillance and chronic stress responses), resulting in a threshold effect where risk rises sharply following a nadir in inflammatory balance.^[37,38]^ Similarly, SIRI (neutrophil × monocyte/ lymphocyte) extends this balance to include monocyte-derived macrophages. During COPD, airway macrophages accumulate, displaying a more pro-inflammatory phenotype and heightened reactive oxygen species release, while their pathogen-phagocytosing capacity diminishes.^[39]^ NPAR, which incorporates neutrophil percentage and serum albumin concentration, serves as a composite indicator of systemic immune-inflammation status and nutritional status; reduced serum albuminis associated with clinical deterioration in COPD patients.^[40]^

However, the precise shapes and inflection points of these associations warrant cautious interpretation, as RCS identifies non-linearity but does not delineate specific trajectories. From a clinical standpoint, the non-linear associations of NLR, NPAR, and SIRI suggest potential inflection points that may inform risk stratification thresholds for screening or therapeutic intervention.

Integration of the aforementioned indices via ML significantly enhances the prognostic performance of conventional models. The NPAR-based NPARTEST model exhibited the optimal performance in terms of discriminative ability (AUROC = 0.828), calibration (MCC = 0.502), and overall predictive efficacy, underscoring the critical role of platelet-mediated pathways in the pathophysiology of COPD. This finding aligns with recent research investigating platelet activation and endothelial dysfunction in COPD-associated thrombotic events. Methodologically, the hybrid feature selection strategy (Boruta algorithm coupled with RFE) effectively mitigated multicollinearity and circumvented model bias induced by variable redundancy, a common limitation in traditional ML studies. However, the cross-sectional study design constrains the robustness of causal inferences, and the generalizability of the study conclusions is limited by the homogeneous population characteristics of the US NHANES cohort.

This study used cross-sectional NHANES data, limiting causal inference between inflammatory markers and mortality. Although indices like NPAR were significantly associated with mortality, it is unclear whether marker elevations precede disease progression or reflect advanced disease. Longitudinal studies are needed to confirm their predictive value. Single-time-point measurements may miss dynamic changes, such as those during acute exacerbations or after treatment. Additionally, NHANES overrepresents non-Hispanic White individuals, limiting generalizability to populations with different genetic, environmental, or healthcare profiles. Future studies should validate these findings in more diverse cohorts – including Black, Hispanic, Asian, and Indigenous groups – to ensure consistent performance across populations. The short follow-up period also limited assessment of long-term effects. Longer prospective studies are required to fully evaluate the sustained impact of inflammation on mortality and its potential as an early biomarker.

## 5. Conclusion

This study demonstrates that systemic inflammatory indices, particularly the neutrophil-platelet-albumin ratio and NHR, exhibit significant nonlinear associations with all-cause mortality in COPD patients. By integrating ML algorithms, we developed the NPARTEST model, which outperformed conventional biomarkers in risk stratification. These findings highlight the potential of cost-effective, routine blood-based indices to enhance prognostic accuracy in COPD management. Future validation in diverse populations is warranted to confirm clinical utility.

## Acknowledgments

The authors would like to express their sincere gratitude to the National Center for Health Statistics (NCHS) for providing access to the NHANES database. We thank all participants of the NHANES survey for their invaluable contributions to this research.

## Author contributions

**Data curation:** Lan Cui, Yichao Zhu.

**Methodology:** Yichao Zhu.

**Resources:** Lan Cui.

**Supervision:** Haohao Qian.

**Writing – review & editing:** Hongyang Xu.

**Writing – original draft:** Lan Cui.
